# IL-1α Promotes Cancer Cell Migration and Is a Potential Prognostic Marker in Oral Squamous Cell Carcinoma

**DOI:** 10.3390/cancers17172781

**Published:** 2025-08-26

**Authors:** Rei Fukui, Shouhei Ogisawa, Akiko Yamada, Masatake Asano

**Affiliations:** 1 Department of Pathology, Nihon University School of Dentistry, 1-8-13 Kanda-Surugadai, Chiyoda-ku, Tokyo 101-8310, Japan; 2 Department of Advanced Oral and Maxillofacial Surgery, Kanagawa Dental University, Yokohama Clinic, 3-31-6 Tsuruya-cho, Kanagawa-ku, Yokohama 221-0835, Kanagawa, Japan

**Keywords:** cancer cell migration, IL-1α, oral squamous cell carcinoma

## Abstract

Background/Objectives: Interleukin-1 alpha (IL-1α) has been linked to tumor progression in various cancer types; however, its role in oral cancer pathogenesis remains largely unexplored. This study aimed to investigate the clinical significance of IL-1α expression in oral squamous cell carcinoma (OSCC), with a specific focus on its role in modulating cancer cell phenotype. Methods: Primary OSCC tissue specimens were collected from 104 patients and subjected to immunohistochemical analysis to assess IL-1α expression. OSCC cell lines were cultured for functional assays, and their protein and mRNA expressions were verified using western blotting and real-time polymerase chain reaction, respectively. The effects of IL-1α expression on OSCC cell proliferation, migration, and gene expression were subsequently examined. Results: IL-1α expression varied among OSCC tissues and cell lines both in vivo and in vitro. Notably, siRNA-mediated suppression of IL-1α in HSC3 cells impaired migration while leaving proliferation unaffected, highlighting its functional role in promoting cancer cell motility. Conclusions: Our in vivo and in vitro findings revealed varied IL-1α expression in OSCC and its association with a motile phenotype, suggesting that case-specific IL-1α assessment could hold prognostic value in oral cancer.

## Simple Summary

Oral squamous cell carcinoma (OSCC) is a prevalent and aggressive malignancy with a high risk of local invasion and recurrence. Interleukin-1 alpha (IL-1α), a proinflammatory cytokine, has been implicated in the progression of various cancers, but its role in OSCC remains poorly understood. In this study, we investigated IL-1α expression in human OSCC tissues and cell lines and examined its relationship with malignant cell behavior. We found that IL-1α was highly expressed at the invasive tumor fronts and in budding cancer cell clusters. Functional assays showed that IL-1α knockdown significantly impaired cancer cell migration without affecting their proliferation. These findings suggest that IL-1α contributes to the invasive potential of OSCC and may serve as a prognostic biomarker for this disease. Targeting IL-1α signaling may provide a novel therapeutic strategy for suppressing OSCC progression.

## 1. Introduction

Oral cancer is the most common type of head and neck malignancy and ranks as the sixth most prevalent cancer globally. Among its subtypes, oral squamous cell carcinoma (OSCC) is the most frequently diagnosed [1], and its surgical management often requires extensive resection of oral tissues essential for functions such as mastication, deglutition, and speech, thereby significantly impairing patients’ quality of life. Therefore, preventing the progression and spread of OSCC is imperative.

Cancer and stromal cells in the tumor microenvironment, which play a vital role in cancer progression, secrete high concentrations of interleukin-1 alpha (IL-1α) under conditions such as hypoxia and nutrition deprivation. IL-1α is a potent proinflammatory cytokine that mediates inflammation, microbial invasion, and tissue injury, and it is expressed in various immune cells, including macrophages and monocytes. It also acts as a mediator of inflammatory responses, inducing inflammation via IL-1R1-dependent pathways upon release into the tumor microenvironment [2,3]. This causes persistent inflammation and stimulates the production of other proinflammatory cytokines, such as IL-6, IL-8, and TNF-α. The persistent inflammatory microenvironment leads to the deterioration of the pathological condition.

IL-1α-neutralizing antibodies are currently employed in the treatment of autoimmune diseases, such as rheumatoid arthritis, as they alleviate joint inflammation via modulation of IL-1α concentrations. Elevated IL-1α expression has been associated with poor prognosis [4,5], and clinical studies by León et al. [6] further demonstrated that high expression of IL-1α in cancer cells correlates with an increased risk of distant metastasis in patients with head and neck cancers [4,5,6]. Therefore, regulating IL-1α concentrations in the cancer microenvironment is imperative. Although recent studies have reported associations between IL-1α and the pathophysiology of various carcinomas [7,8,9,10,11,12,13,14], its role in oral cancer pathogenesis remains largely unexplored. The present study aims to investigate the clinical significance of IL-1α expression in OSCC and its importance in disease pathology through immunohistochemical analysis complemented by in vitro molecular assessments. This study reports the first assessment of IL-1α expression in human clinical specimens of OSCC using immunohistochemistry.

## 2. Materials and Methods

All experimental procedures were conducted in accordance with the ethical standard of the amended Declaration of Helsinki and were approved by the Ethics Committee of Nihon University (Registry No. EP22D002, Approval date: 14 April 2022).

### 2.1. Clinical Specimens

Primary OSCC specimens were obtained from 104 patients treated at the Dental Hospital of Nihon University between 2017 and 2022. The specimens included SCCs of the tongue, gingiva, buccal mucosa, palate, and floor of the mouth. Tissues used for immunohistochemical analysis were collected via biopsy or surgical resection, fixed in 10% buffered formalin solution for 1–3 days, and then embedded in paraffin. The detailed clinical characteristics of the 104 patients in this study are presented in Table 1.

### 2.2. Immunohistochemical Analyses

Immunohistochemical staining was performed on formalin-fixed, paraffin-embedded human OSCC tissue sections. A rabbit polyclonal anti-IL-1α antibody (1:100 dilution; ab227482, Abcam, CA, USA) was used as the primary antibody. Antigen retrieval was performed by treating the sections with HISTOFINE antigen retrieval buffer (pH = 9.0; 415211, Nichirei Bioscience, Tokyo, Japan) for 20 min at 95 °C according to the manufacturer’s protocol. The EnVision + Dual Link System-HRP (Dako, CA, USA) was used as the secondary antibody, and coloration was performed using a 3,3′ -diaminobenzidine substrate (Dako, CA, USA). The immunohistochemical evaluation was performed by two independent pathologists (R.F. and A.Y.) who were blinded to the clinicopathological data of each case. IL-1α expression in tumor cells was evaluated semi-quantitatively using a novel three-tier scoring system based on staining intensity and the percentage of positive cells. The staining was classified as: negative (no staining), low (<30% of cells with weak staining), or high (>30% of tumor cells with moderate cytoplasmic staining). This scoring system was designed for simplicity and reproducibility in routine diagnostics and differs from previously reported dual nuclear/cytoplasmic scoring systems [15]. Cutoff values were based on internal validation data.

### 2.3. Cell Culture

Five head and neck SCC cell lines (HSC2, HSC3, HSC4, Ca9-22, and UM-SCC6) were obtained from the oral cavity and used for cell culture. Primary normal human dermal fibroblasts (NHDFs) from adult donors were also included in the study. HSC3, Ca9-22, and UM-SCC6 cell lines were provided by Dr. Kyoko Fujiwara, and HSC2 and HSC4 cell lines were obtained from our laboratory. NHDFs from adult donors were purchased from PromoCell (C-12302, Heidelberg, Germany) and cultured in Fibroblast Growth Medium 2 (PromoCell) according to the manufacturer’s protocol. The culture conditions varied by cell line. HSC3 cells were cultured in Minimum Essential Medium (MEM) (Nacalai Tesque, Inc., Kyoto, Japan) containing 10% fetal bovine serum (FBS) (Sigma-Aldrich, St. Louis, MO, USA) and antibiotics (168-23191, Wako, Osaka, Japan). Ca9-22 cells were maintained in MEM containing 10% FBS, antibiotics, and 0.6 g/L glutamine (13004-02, Nacalai Tesque, Inc., Kyoto, Japan). HSC2 and HSC4 cells were maintained in Dulbecco’s Modified Eagle Medium (DMEM) (Thermo Fisher Scientific, Waltham, MA, USA) containing 10% FBS and antibiotics. UM-SCC6 cells were cultured in DMEM containing 15% FBS and antibiotics.

### 2.4. Cell Proliferation and Wound-Healing Assay

HSC3 cells were seeded into a 96-well plate at a density of 1.0 × 10^3^ cells/well to examine the effect of IL-1α expression on the proliferation of OSCC cell lines. The cells were transfected with si-IL-1α (with or without [control]) at 12 h after seeding and incubated for 24, 48, and 72 h. Cell proliferation was measured using the WST-8 reagent (Nacalai Tesque, Inc., Kyoto, Japan) according to the manufacturer’s instructions.

The cells (1.4 × 10^4^ cells) were seeded into culture inserts (Ibidi, ib80209; NIPPON Genetics Co., Ltd., Tokyo, Japan) for the wound-healing assay following the procedure outlined in a previous report [16]. siRNA was transfected after the cells adhered to the wells. The culture inserts were removed upon reaching confluence, and the cells were cultured in their respective media. Cell migration was assessed by measuring the movement of cells into the scratched area. Representative images (×10) of wound closure were captured at 0 and 14 h using an All-in-One Fluorescence Microscope (BZ-X810) and analyzed using a BX-Z810 analyzer (KEYENCE, Osaka, Japan).

### 2.5. Enzyme-Linked Immunosorbent Assay

The OSCC cells were seeded or transfected with siRNA and cultured in serum-free medium for 24 h. The concentrations of IL-1α in the culture medium were determined using commercially available enzyme-linked immunosorbent assay (ELISA) kits (IL-1α: R&D Systems, Minneapolis, MN, USA) according to the manufacturer’s instructions. Total protein concentrations in the culture supernatant were measured using the Pierce BCA Protein Assay Kit (Thermo Scientific, Rockford, IL, USA).

### 2.6. Real-Time Polymerase Chain Reaction

The cells were seeded into 6-well plates and cultured. Total RNA was isolated using the RNeasy Mini Kit (QIAGEN, Valencia, CA, USA), and RNA concentration was determined using NanoDrop 1000 (Thermo Fisher Scientific, Wilmington, DE, USA). Complementary DNA (cDNA) was synthesized from 800 ng of DNase-treated total RNA using the PrimeScript RT Master Mix (Takara Bio, Shiga, Japan). The resulting cDNA was analyzed via real-time PCR using TB Green Premix Ex Taq II (Takara Bio). The PCR reactions were performed in a total volume of 25 μL, which comprised 12.5 μL of TB Green Premix Ex Taq II, 0.5 μL (10 μM) of each primer (Table 1), 9.5 μL of distilled water, and 2 μL of cDNA template. PCR was performed using the Thermal Cycler Dice Real-Time System II (Takara Bio) under the following cycling conditions: 35 cycles at 95 °C for 5 s and 60 °C for 20 s. All real-time PCR experiments were performed in triplicate, and the amplification specificity was verified using melting curve analysis. The target mRNA levels for IL-1α were calculated using the ΔCt method with β-actin as the internal control. The primer sequences are listed in Table 1.

### 2.7. Statistical Analysis

The data represent the results of three or four independent experiments, each with samples tested in triplicate. They are expressed as the mean ± standard deviation. Group differences were analyzed using one-way analysis of variance followed by Tukey’s multiple comparison test or an unpaired t-test. Statistical analyses were performed using GraphPad Prism Version 10.2.3 (GraphPad Software, Boston, MA, USA). Differences were considered statistically significant at *p* < 0.05.

## 3. Results

### 3.1. IL-1α Expression in Human OSCC Surgical Specimens

**Figure 1:**
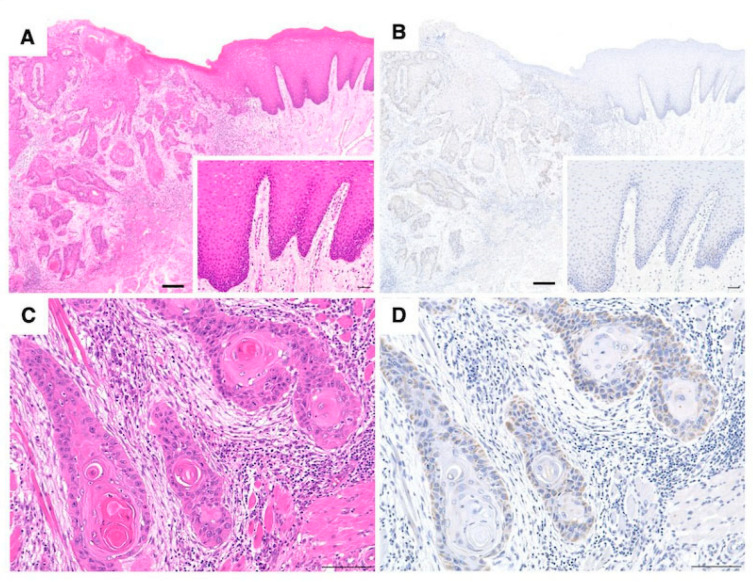
Histology of human OSCC. Distribution of H&E staining (**A**,**B**) and IHC of IL-1α (**C**,**D**). Tumor tissues stained brown (positive) while normal tissues did not stain (negative) for IL-1α (**B**). Scale bar = 200 µm. Inset: Magnified image of the normal squamous epithelium. Scale bar = 50 µm. H&E staining (**C**) and IHC (**D**) for IL-1α at the tumor invasion front of human OSCC. Scale bar = 100 µm.

IL-1α expression in human clinical specimens was investigated using IHC staining. The tumor tissue stained positive brown, whereas the normal tissues were negative for IL-1α (Figure 1A,B). IHC staining revealed strong IL-1α expression in basal-like cells at the margins of the differentiated SCC tumor nests, while keratinized tumor cells distant from the stroma were negative for IL-1α (Figure 1C,D). Among the 104 human cases evaluated, 44 (42.3%) were positive for IL-1α. The correlations between IL-1α expression and clinicopathological features (patient age, sex, site of presentation, differentiation, presence or absence of lymph node metastasis, and tumor size) were also investigated. A trend suggesting lower differentiation was associated with higher IL-1α expression was observed (Table 2), but it was not statistically significant.

### 3.2. Clinicopathological Significance of IL-1α Expression in OSCC

**Figure 2:**
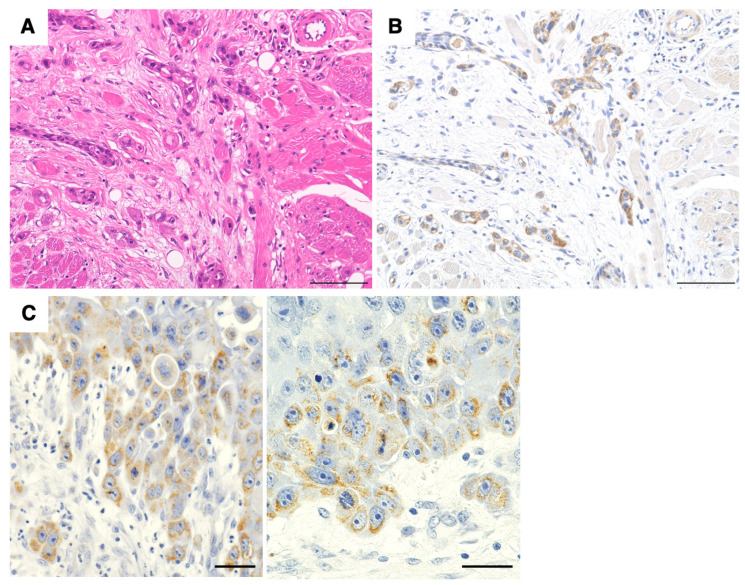
Correlation between IL-1α and invasiveness. H&E (**A**) and IHC (**B**,**C**) staining for IL-1α. IHC shows strong positivity for IL-1α staining in budding and small cancer cell clusters at the invasion front (**C**). (**A**,**B**) Scale bar = 100 µm. (**C**) Scale bar = 50 µm.

The expression of IL-1α was also prominent in elongated, cord-like clusters of cancer cells invading the surrounding tissue (Figure 2A,B). It was also observed in basaloid cells at the invasion front, such as budding-like foci that spread out of the base, individual cells, and small clusters of 2–3 cancer cells (Figure 2C). These findings suggest that IL-1α expressed by cancer cells is associated with a phenotype that exacerbates the disease.

### 3.3. IL-1α Synthesis Levels Differed Among OSCC Surgical Specimens and Culture Cell Lines

**Figure 3:**
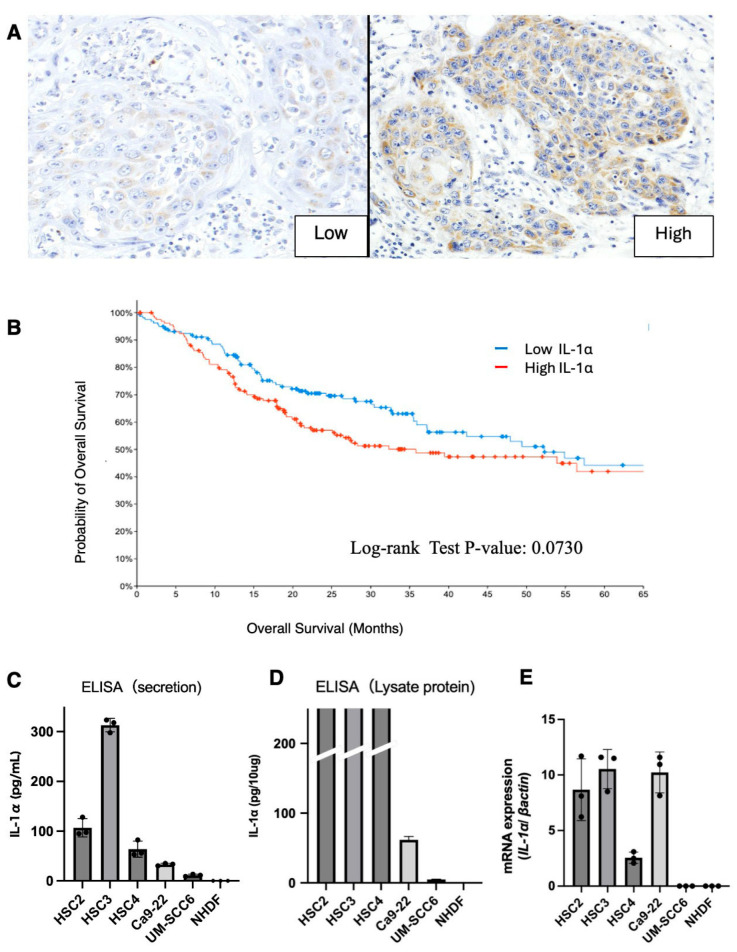
Criteria for the evaluation of IL-1α staining (**A**) displayed in medium enlargement. Kaplan–Meier curve for overall survival in relation to IL-1α expression derived using cBioPortal (TCGA, PanCancer Atlas). (**B**) Log-rank test was used to calculate significance. Secreted (**C**) and intracellular (**D**) IL-1α in OSCC cell lines. IL-1a expression was analyzed in the media and cell lysates (*n* = 3) using ELISA. The expression levels of IL-1α mRNAs were determined using real-time PCR (**E**).

The assessment of IL-1α expression using immunostaining of human clinical specimens has not been performed. Therefore, we established criteria to determine the intensity and assess the properties of the staining. Representative staining images are provided in Figure 3A. Cases with low and high staining were considered positive. A comprehensive cross-sectional analysis of cancer databases (cBioPortal) revealed that higher IL-1α expression in cancer cells was associated with a lower 5-year survival rate (Figure 3B).

We performed in vitro experiments focusing on OSCC cell lines and primary NHDFs to investigate IL-1α expression in cultured cells. All OSCC cell lines secreted high concentrations of IL-1α into the culture supernatant, although at varying degrees. Conversely, IL-1α secretion and expression in the NHDFs were negligible and below the detection range of ELISA (Figure 3C). IL-1α expression levels in the cell lysates were high for the HSC2, HSC3, and HSC4 cell lines (Figure 3D); they were independent of the concentrations secreted. Relatively high levels of mRNA expression were also observed in each cell line; however, neither mRNA nor protein expression was confirmed in NHDF (Figure 3E). Among the OSCC lines, HSC3 exhibited the highest IL-1α expression levels, whereas UM-SCC6 cells had the lowest, despite both being derived from tongue cancers.

### 3.4. Inhibition of IL-1α Expression Suppressed Cancer Cell Migration

**Figure 4:**
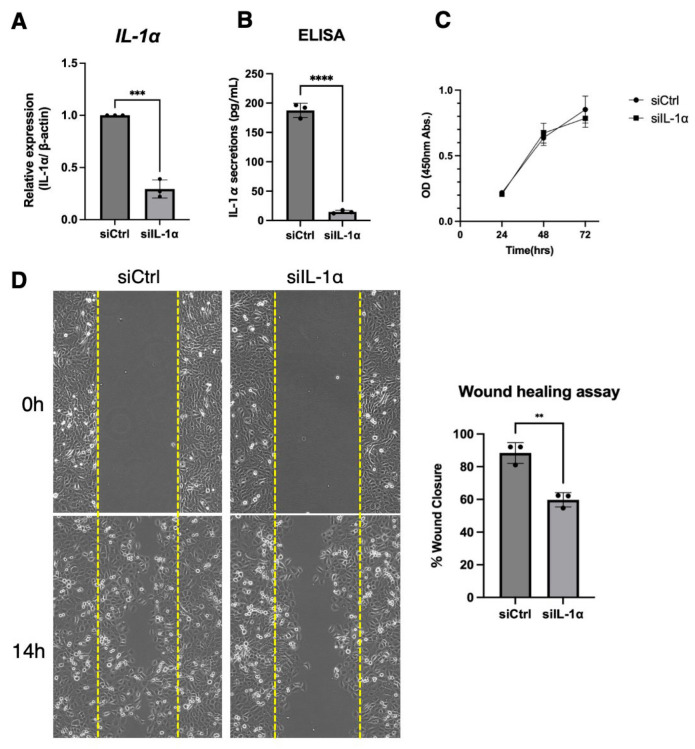
Characteristics of cells with downregulated IL-1α expression. IL-1α mRNA expression, assessed with real-time PCR (**A**), and IL-1α secretion, assessed with ELISA (**B**), were markedly suppressed. Cells were transfected with negative control siRNA (siCtrl) or IL-1α-specific siRNA (siIL-1α) for 24 h. ***: *p* < 0.0005, ****: *p* < 0.0001. Transfected cells were seeded and examined for cell proliferation at 24, 48, and 72 h (**C**) and migration at 14 h (**D**). The yellow dotted line indicates the starting point of the wound healing assay. **: *p* < 0.05.

The effects of IL-1α expression on the HSC3 cell line were also investigated. After 24 h of siRNA transfection, the cancer cells exhibited decreased IL-1α mRNA expression and secretion (Figure 4A,B). The effect of IL-1α knockdown on the cancer cell phenotype was subsequently evaluated. Cell proliferation in pretreated cells was not significantly affected by IL-1α knockdown (Figure 4C). However, the wound healing assay demonstrated significant inhibition of migration in OSCC cell lines following IL-1α suppression (Figure 4D). These results suggest that inhibiting IL-1α expression in cancer cells impairs their migratory ability.

## 4. Discussion

IL-1α is constitutively expressed in various cell types and was historically regarded as a passive “alarmin” released from necrotic cells [10]. More recent studies suggest that IL-1α can also be activated by sublethal damage, such as oxidative stress and DNA injury [16], and plays roles in both tumor progression and anti-tumor immunity within the tumor microenvironment [17]. In the present study, we observed heterogeneous IL-1α expression in OSCC tissues, providing new insights into its biological functions.

In our cohort, approximately 40% of OSCC specimens exhibited high IL-1α expression, with marked variation even within the same tumor. This heterogeneity may be influenced by tumor subtype; for example, basaloid OSCC—characterized by higher invasiveness and epithelial–mesenchymal transition (EMT) potential [18,19]—showed a tendency toward stronger IL-1α immunoreactivity. Epigenetic and transcriptional mechanisms, including promoter methylation and upstream signaling alterations [20], may also contribute. Furthermore, IL-1α upregulation could reflect a proinflammatory microenvironment enriched in cancer-associated fibroblasts (CAFs) and immune infiltrates [21,22,23]. These patterns support the potential utility of IL-1α expression profiling as a stratification biomarker for patients with OSCC [4].

Functionally, IL-1α knockdown significantly suppressed OSCC cell migration without affecting proliferation, reinforcing its role in motility rather than general growth. High IL-1α expression was localized to invasive fronts, tumor budding foci, and small cell clusters—histological features often linked to EMT and local invasion. Given these observations, IL-1α may influence EMT regulation and warrant further investigation.

Consistent with previous reports that IL-1 signaling enhances migration via MMP-9 and NF-κB activation [11,22,24,25], our CRISPR-Cas9 knockout experiments confirmed that recombinant IL-1α restored migration capacity in IL-1α–deficient cells. This demonstrates that both endogenous and exogenous IL-1α can enhance the motility of OSCC. Moreover, co-culture experiments indicated that IL-1α can enhance stromal activation and inflammatory cytokine production, reinforcing a tumor-promoting microenvironment [21,22,23,24,25,26,27].

This study has limitations. We did not directly assess IL-1α expression in CAFs or immune cells, and detailed mechanistic analyses such as spatial transcriptomics or single-cell RNA sequencing remain for future work. Nonetheless, our in vivo and in vitro findings consistently demonstrate a link between IL-1α and enhanced cell migration in OSCC. Prior studies have shown that IL-1α blockade can attenuate tumor-promoting inflammation [2,5,6,9,28], suggesting therapeutic potential. Targeting IL-1α signaling—via neutralizing antibodies or pathway inhibitors—could simultaneously modulate tumor cells and stromal fibroblasts [29], disrupting the inflammatory crosstalk that drives OSCC progression.

## 5. Conclusions

This is the first study to report high IL-1α expression specifically localized to clinically relevant tumor regions, such as the invasive front and budding-like structures, in OSCC. The level of expression of IL-1α in OSCC cells may serve as an important prognostic factor.

## Figures and Tables

**Table 1:** Polymerase chain reaction primers used in this study.

Target		Primers
IL-1α	F	5′-AACCAGTGCTGCTGAAGG-3′
	R	5′-TTCTTAGTGCCGTGAGTTTCC-3′
β-actin	F	5′-CACCATTGGCAATGAGCGGTTC-3′
	R	5′-AGGTCTTTGCGGATGTCCACGT-3′

**Table 2:** Characteristics of the patients included in this study.

Characteristics		IL-1α Positive		IL-1α Negative		*p* Value	χ^2^
Numbers of patients		44	42.3%	60	57.7%		
Age (years)	Median	66.5					
	Range	(22–91)					
Sex	Male	29	27.9%	40	38.5%	1	0
	Female	15	14.4%	20	19.2%		
Tumor Location	Tongue	23	52.3%	32	53.3%	0.1042	9.1424
	Gingiva	13	29.5%	19	31.7%		
	Buccal mucosa	1	2.3%	6	10.0%		
	Floor of the mouth	3	6.8%	2	3.3%		
	Palate	4	9.1%	0	0.0%		
	Lip	0	0.0%	1	1.7%		
Differentiation	G1	22	50.0%	39	65.0%	0.0591	3.835
	G2	15	34.1%	19	31.7%		
	G3	7	15.9%	2	3.3%		
N category	cN0	31	70.5%	33	55.0%	0.5735	0.0835
	N1≦	7	15.9%	12	20.0%		
	Unknown	6	13.6%	15	25.0%		
Tumor size	2 cm≧	21	47.7%	38	63.3%	0.1655	1.923
	2 cm≦	23	52.3%	22	36.7%		

## Data Availability

The datasets used in this study are available from the corresponding author upon reasonable request.

## Abbreviations

The following abbreviations are used in this manuscript:

CAF	Cancer-associated fibroblasts
DMEM	Dulbecco’s Modified Eagle Medium
ELISA	Enzyme-linked immunosorbent assay
EMT	Epithelial-Mesenchymal transition
FBS	Fetal bovine serum
IL-1α	Interleukin-1 alpha
MEM	Minimum essential medium
NHDF	Normal human dermal fibroblasts
OSCC	Oral squamous cell carcinoma
